# Avoidable errors in the modelling of outbreaks of emerging pathogens, with special reference to Ebola

**DOI:** 10.1098/rspb.2015.0347

**Published:** 2015-05-07

**Authors:** Aaron A. King, Matthieu Domenech de Cellès, Felicia M. G. Magpantay, Pejman Rohani

**Affiliations:** 1 Department of Ecology & Evolutionary Biology, University of Michigan, Ann Arbor, MI 48109, USA; 2 Center for the Study of Complex Systems, University of Michigan, Ann Arbor, MI 48109, USA; 3 Department of Mathematics, University of Michigan, Ann Arbor, MI 48109, USA; 4 Fogarty International Center, National Institutes of Health, Bethesda, MD 20892, USA

**Keywords:** Ebola virus disease, forecast, emerging infectious disease

## Abstract

As an emergent infectious disease outbreak unfolds, public health response is reliant on information on key epidemiological quantities, such as transmission potential and serial interval. Increasingly, transmission models fit to incidence data are used to estimate these parameters and guide policy. Some widely used modelling practices lead to potentially large errors in parameter estimates and, consequently, errors in model-based forecasts. Even more worryingly, in such situations, confidence in parameter estimates and forecasts can itself be far overestimated, leading to the potential for large errors that mask their own presence. Fortunately, straightforward and computationally inexpensive alternatives exist that avoid these problems. Here, we first use a simulation study to demonstrate potential pitfalls of the standard practice of fitting deterministic models to cumulative incidence data. Next, we demonstrate an alternative based on stochastic models fit to raw data from an early phase of 2014 West Africa Ebola virus disease outbreak. We show not only that bias is thereby reduced, but that uncertainty in estimates and forecasts is better quantified and that, critically, lack of model fit is more readily diagnosed. We conclude with a short list of principles to guide the modelling response to future infectious disease outbreaks.

## 1. Introduction

The success of model-based policy in response to outbreaks of bovine spongiform encephelopathy [1] and foot-and-mouth disease [2,3] established the utility of scientifically informed disease transmission models as tools in a comprehensive strategy for mitigating emerging epidemics. Increasingly, the expectation is that reliable forecasts will be available in real time. Recent examples in which model-based forecasts were produced within weeks of the index case include severe acute respiratory syndrome (SARS; [4,5]), pandemic H1N1 influenza [6], cholera in Haiti and Zimbabwe [7], Middle East respiratory syndrome (MERS; [8]), and lately, Ebola virus disease (EBVD) in West Africa [9,10]. In the early stages of an emerging pathogen outbreak, key unknowns include its transmission potential, the likely magnitude and timing of the epidemic peak, total outbreak size, and the durations of the incubation and infectious phases. Many of these quantities can be estimated using clinical and household transmission data, which are, by definition, rare in the early stages of such an outbreak. Much interest, therefore, centres on estimates of these quantities from incidence reports that accumulate as the outbreak gathers pace. Such estimates are obtained by fitting mathematical models of disease transmission to incidence data.

As is always the case in the practice of confronting models with data, decisions must be made as to the structure of fitted models and the data to which they will be fit. Concerning the first, in view of the urgency of policy demands and paucity of information, the simplest models are, quite reasonably, typically the first to be employed. With even the simplest models, such as the classical susceptible–infected–recovered (SIR) model, the choice of data to which the model is fit can have significant implications for science and policy. Here, we explored these issues using a combination of inference on simulated data and on actual data from an early phase of the 2013–2015 West Africa EBVD outbreak. We find that some of the standard choices of model and data can lead to potentially serious errors. Since, regardless of the model choice, all model-based conclusions hinge on the ability of the model to fit the data, we argue that it is important to seek out evidence of model misspecification. We demonstrate an approach based on stochastic modelling that allows straightforward diagnosis of model misspecification and proper quantification of forecast uncertainty.

## 2. Deterministic models fit to cumulative incidence curves: a recipe for error and overconfidence

**Figure 1.**
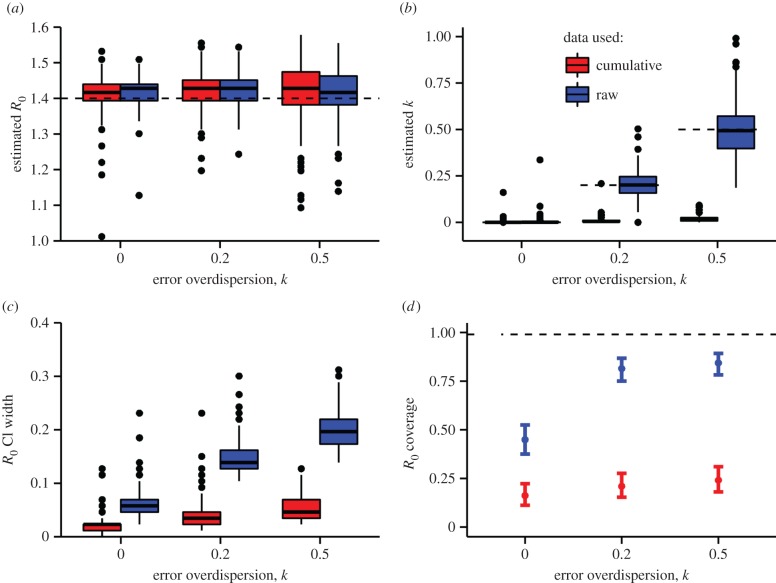
Results from simulation study fitting deterministic models to stochastically simulated data. Five hundred simulated datasets of length 39 weeks were generated by the stochastic model described in §5 at each of three levels of the measurement error overdispersion parameter, *k*. The deterministic model was fit to both raw (blue) and accumulated (red) incidence data. (*a*) Estimates of *R*_0_. True value used in generating the data is shown by the dashed line. (*b*) Estimates of error overdispersion, *k*. (*c*) Widths of nominal 99% profile-likelihood confidence intervals (CI) for *R*_0_. (*d*) Actual coverage of the CI, i.e. probability that the true value of *R*_0_ lay within the CI. Ideally, actual coverage would agree with nominal coverage (99%, dashed line).

An inexpensive and therefore common strategy is to formulate deterministic transmission models and fit these to data using least squares or related methods. These approaches seek parameters for which model trajectories pass as close to the data as possible. Because, in such an exercise, the model itself is deterministic, all discrepancies between model prediction and data are in effect ascribed to measurement error. Implicitly, the method of least squares assumes that these errors are independent and normally distributed, with a constant variance. This assumption can be replaced without difficulty by more realistic assumptions of non-normal errors and, in particular, an error variance that depends on the mean. As for the data to be fit, many have opted to fit model trajectories to cumulative case counts. The incompatibility of this choice with the assumptions of the statistical error model has been pointed out previously [11–13]. In particular, the validity of the statistical estimation procedure hinges on the independence of sequential measurement errors, which is clearly violated when observations are accumulated through time (see electronic supplementary material, appendix B). To explore the impact of this violation on inferences and projections, we performed a simulation study in which we generated data using a stochastic model, then fit the corresponding deterministic model to both raw and cumulative incidence curves. We generated 500 sets of simulated data at each of three different levels of measurement noise. For each dataset, we estimated model parameters, including transmission potential (as quantified by the basic reproduction number, *R*_0_) and observation error overdispersion (as quantified by the negative binomial overdispersion parameter, *k*). Full details of the data generation and fitting procedures are given in electronic supplementary material, appendix A. The resulting parameter estimates are shown in figure 1.

Recognizing that quantification of uncertainty is prerequisite to reliable forecasting, we computed parameter estimate confidence intervals and investigated their accuracy. Figure 1*a* shows that, in estimating *R*_0_, one finds considerable error but little evidence for bias, whether raw or cumulative incidence data are used. Although in general one expects that violation of model assumptions will introduce some degree of bias, in this case since both the raw and cumulative incidence curves generically grow exponentially at a rate determined by *R*_0_, estimates of this parameter are fairly accurate, *on average*, when data are drawn, as here, from the early phase of an outbreak. Figure 1*b* is the corresponding plot of estimated overdispersion of measurement noise. Using the raw incidence data, one recovers the true observation variability. When fitted to cumulative data, however, the estimates display extreme bias: far less measurement noise is needed to explain the relatively smooth cumulative incidence. The data superficially appear to be in very good agreement with the model.

To quantify the uncertainty in the parameter estimates, we examined the confidence intervals. The nominal 99% profile-likelihood confidence interval widths for *R*_0_ are shown in figure 1*c*. When the model is fit to the simulated data, increasing levels of measurement error lead to increased variance in the estimates of *R*_0_. However, the confidence interval widths are far smaller when the cumulative data are used, superficially suggesting a higher degree of precision. This apparent precision is an illusion, however, as figure 1*d* shows. This figure plots the achieved coverage (probability that the true parameter value lies within the estimated confidence interval) as a function of the magnitude of measurement error and the choice of data fitted. Given that the nominal confidence level here is 99%, it is disturbing that the true coverage achieved is closer to 25% when cumulative data are used.

When a deterministic model is fit to cumulative incidence data, the net result is a potentially quite over-optimistic estimate of precision, for three reasons. First, failure to account for the non-independence of successive measurement errors leads to an underestimate of parameter uncertainty (figure 1*c*). Second, as seen in figure 1*b*, the variance of measurement noise will be substantially underestimated. Finally, because the model ignores environmental and demographic stochasticity—treating the unfolding outbreak as a deterministic process—forecast uncertainty will grow unrealistically slowly with the forecast horizon. We elaborate on the last point in §4.

## 3. Stochastic models fit to raw incidence data: feasible and transparent

The incorporation of demographic and/or environmental stochastic processes into models allows, on the one hand, better fits to the trends and variability in data and, on the other, improved ability to diagnose lack of model fit [14]. We formulated a stochastic version of the susceptible–exposed–infectious–recovered (SEIR) model as a partially observed Markov process and fit it to actual data from an early phase of the 2013–2015 West Africa EBVD outbreak. We estimated parameters by maximum likelihood, using sequential Monte Carlo to compute the likelihood and iterated filtering to maximize it over unknown parameters [15]. See electronic supplementary material, appendix B for details.

**Figure 2.**
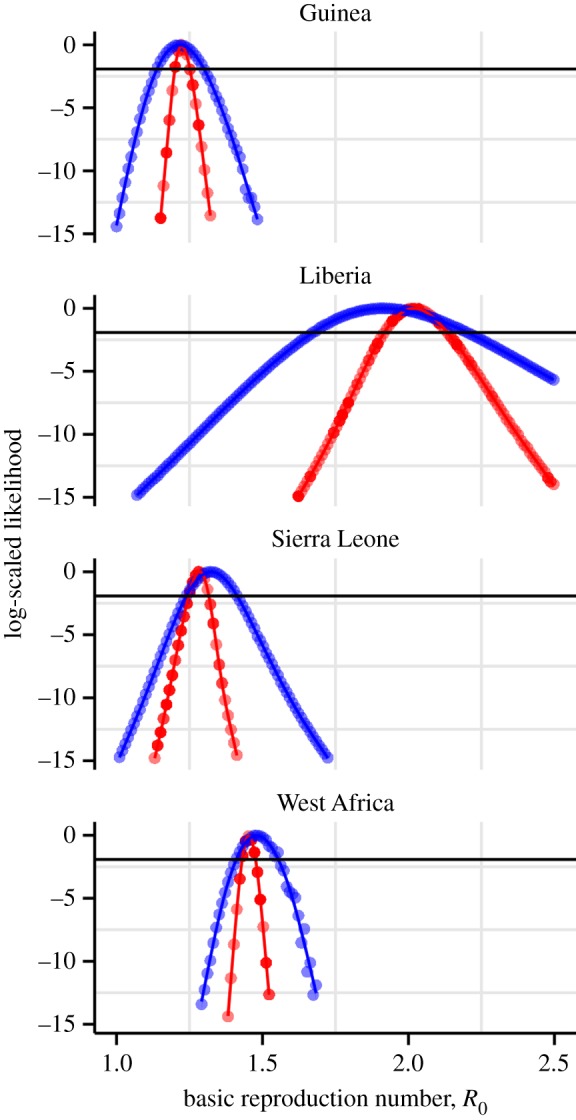
Likelihood profiles for *R*_0_ based on the stochastic model fit to raw data (blue) versus the deterministic model fit to cumulative incidence data (red). Each point represents the maximized log likelihood at each fixed value of *R*_0_ relative to overall maximum. The maximum of each curve is achieved at the maximum-likelihood estimate (MLE) of *R*_0_; the curvature is proportional to estimated precision. The horizontal line indicates the critical value of the likelihood ratio at the 95% CI. While the (improper) use of cumulative data produces relatively small differences in the MLE for *R*_0_, it does produce the illusion of high precision.

Figure 2 shows likelihood profiles over *R*_0_ for country-level data from Guinea, Liberia and Sierra Leone. We also wanted to explore the potential for biases associated with spatial aggregation of the data. Hence, we fit our models to regional data, encompassing all reported cases from the three West African countries just mentioned. In line with the lessons of figure 1*c*, estimated confidence intervals are narrower when the cumulative reports are used. The ‘true’ parameters are, of course, unknown, but, as in the earlier example, this higher precision is probably illusory. The somewhat, but not dramatically, larger confidence intervals that come with adherence to the independent-errors assumption (i.e. with the use of raw incidence data) lead to a quite substantial increase in forecast uncertainty, as we shall see. Finally, the ease with which the stochastic model was fit and likelihood profiles computed testifies to the fact that, in the case of outbreaks of emerging infectious diseases, it is not particularly difficult or time-consuming to work with stochastic models.

**Figure 3.**
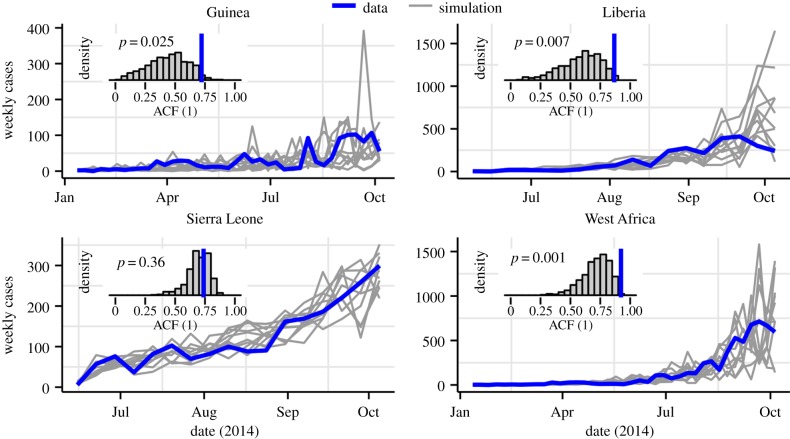
Model diagnostics. The time series plots show the data (blue) superimposed on 10 typical simulations from the fitted model (grey). While the overall trend is captured by the model, the simulations display more high-frequency (week-to-week) variability than does the data. The insets confirm this, showing the autocorrelation function at lag 1 week (ACF(1)) in the data (blue) superimposed on the distribution of ACF(1) in 500 simulations (grey). For Guinea, Liberia and the aggregated regional data (‘West Africa’), the ACF(1) of the data lies in the extreme right tail of the distribution, as quantified by the one-sided *p*-values shown.

We took advantage of the stochastic model formulation to diagnose the fidelity of model to the data. To do so, we simulated 10 realizations of the fitted model; the results are plotted in figure 3. While the overall trends appear similar, the model simulations display greater variability at high frequencies than do the data. To quantify this impression, we computed the correlation between cases at weeks *t* and *t* – 1 (i.e. the autocorrelation function at lag one week, ACF(1)) for both model simulations and data. For Guinea, Liberia, and the region as a whole (‘West Africa’), the observed ACF(1) lies in the extreme right tail of the model-simulated distribution, confirming our suspicion. For Sierra Leone, the disagreement between fitted model and data is not as great, at least as measured by this criterion. These diagnostics caution against the interpretation of the outbreaks in Guinea and Liberia as simple instances of SEIR dynamics, and call for a degree of scepticism in inferences and forecasts based on this model. On the other hand, the Sierra Leone epidemic does appear, by this single metric, to better conform to the SEIR assumptions when the data are aggregated to the country level.

**Figure 4.**
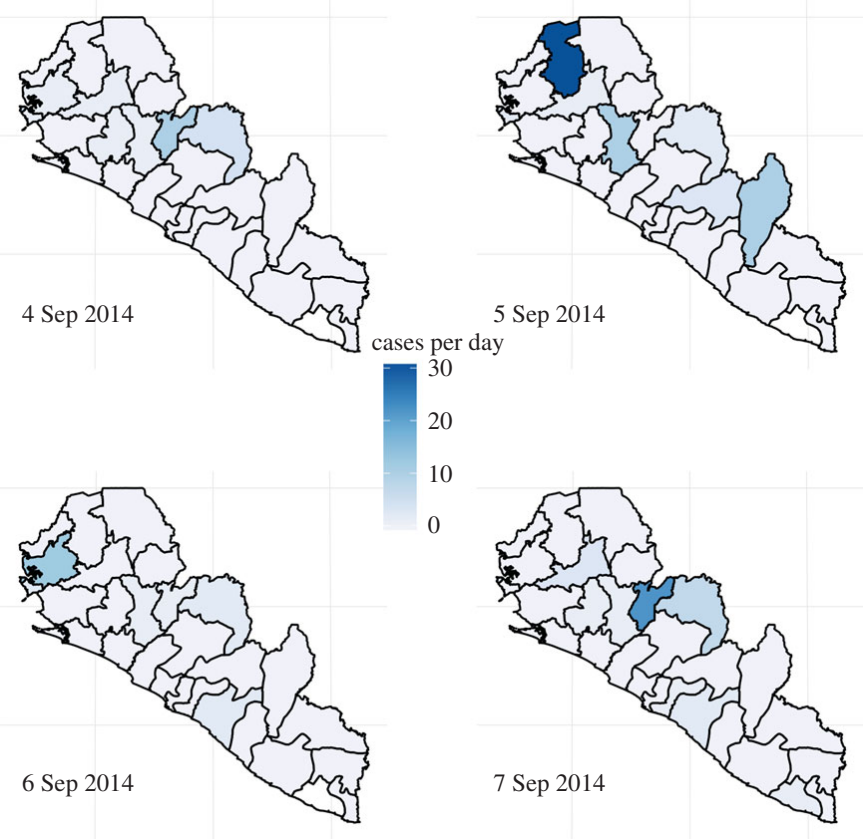
Four consecutive days of Ebola incidence in the republics of Liberia and Sierra Leone. In the outbreak's early stages, the spatio–temporal dynamics are highly erratic, contrary to the predictions of the well-mixed model. (Online version in colour.)

Figure 4 suggests why the present Ebola outbreak might not be adequately described by the well-mixed dynamics of the SEIR model. The erratically fluctuating mosaic of localized hotspots suggests spatial heterogeneity in transmission, at odds with the model's assumption of mass action. As an aside, this heterogeneity hints at control measures beyond the purview of the SEIR model. While the latter might provide more or less sound guidance with respect to eventual overall magnitude of the outbreak and associated demands for hospital beds, treatment centres, future vaccine coverage, etc., the former points to the potential efficacy of movement restrictions and spatial coordination of control measures.

## 4. Discussion

**Figure 5.**
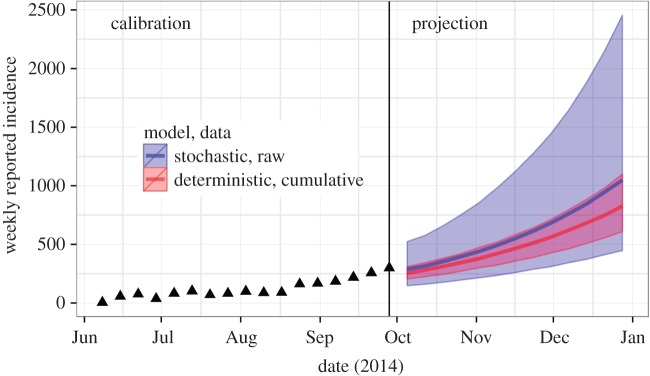
Forecast uncertainty for the Sierra Leone EBVD outbreak as a function of the model used and the data to which the model was fit. The red ribbon shows the median and 95% envelope of model simulations for the deterministic SEIR model fit to cumulative case reports; the blue ribbon shows the corresponding forecast envelope for the stochastic model fit to raw incidence data. The data used in model fitting are shown using black triangles.

To summarize, we have here shown that the frequently adopted approach of fitting deterministic models to cumulative incidence data can lead to bias and pronounced underestimation of the uncertainty associated with model parameters. Not surprisingly, forecasts based on such approaches are similarly plagued by difficult-to-diagnose overconfidence as well as bias. We illustrated this using the SEIR model—in its deterministic and stochastic incarnations—fit to data from the current West Africa EBVD outbreak. Emphatically, we do not here assert that the SEIR model adequately captures those features of the epidemic needed to make accurate forecasts. Indeed, when more severe diagnostic tests are applied (electronic supplementary material, figure B1), it seems less plausible that the Sierra Leone data appear are a sample from the model distribution. Moreover, we have side-stepped important issues of identifiability of key parameters such as route-specific transmissibility, asymptomatic ratio and effective infectious period. Rather, we have purposefully oversimplified, both to better reflect modelling choices often made in the early days of an outbreak and to better focus on issues of statistical practice in the context of quantities of immediate and obvious public health importance, particularly the basic reproduction number and predicted outbreak trajectory. Figure 5 shows projected incidence of EBVD in Sierra Leone under both the deterministic model fit to cumulative incidence data (in red) and the stochastic model fit to raw incidence data (in blue). The shaded ribbons indicate forecast uncertainty. In the deterministic case, the latter is due to the combined effects of estimation error and measurement noise. As we showed above, the first contribution is unrealistically low because serial autocorrelation among measurement errors have not been properly accounted for. The second contribution is also underestimated because of the smoothing effect of data accumulation. Finally, because the model ignores all process noise, it unrealistically lacks dynamic growth of forecast uncertainty. By contrast, the stochastic model fitted to the raw incidence data show much greater levels of uncertainty. Because measurement errors have been properly accounted for, confidence intervals more accurately reflect true uncertainty in model parameters. Because the model accounts for process noise, uncertainty expands with the forecast horizon. Finally, we recall once again that, because the process noise terms can to some degree compensate for model misspecification, it was possible to diagnose the latter, thus obtaining some additional qualitative appreciation of the uncertainty owing to this factor.

The increasingly high expectations placed on models as tools for public policy put an ever higher premium on the reliability of model predictions, and therefore on the need for accurate quantification of the associated uncertainty. The relentless trade-off between timeliness and reliability has with technological advance shifted steadily in favour of more complex and realistic models. Because stochastic models with greater realism, flexibility and transparency can be routinely and straightforwardly fit to outbreak data, there is less and less scope for older, less reliable and more opaque methods. In particular, the practices of fitting deterministic models and fitting models to cumulative case report data are prejudicial to accuracy and can no longer be justified on pragmatic grounds. We propose the following principles to guide modelling responses to current and future infectious disease outbreaks:

(1) models should be fit to raw, disaggregated data whenever possible and never to temporally accumulated data;
(2) when model assumptions, such as independence of errors, must be violated, careful checks for the effects of such violations should be performed;
(3) forecasts based on deterministic models, being by nature incapable of accurately communicating uncertainty, should be avoided; and
(4) stochastic models should be preferred to deterministic models in most circumstances because they afford improved accounting for real variability and increased opportunity for quantifying uncertainty. *Post hoc* comparison of simulated and actual data is a powerful and general procedure that can be used to distinguish model misspecification from real stochasticity.

In closing, we are troubled that screening for lack of model fit is not a completely standard part of modelling protocol. At best, this represents a missed opportunity, as discrepancies between the data and off-the-shelf models may suggest effective control measures. At worst, this can lead to severely biased estimates and, worryingly, overly confident conclusions. Fortunately, effective techniques exist by which such errors can be diagnosed and avoided, even in circumstances demanding great expedition.

## 5. Material and methods

### (a) Data

Weekly case reports in Guinea, Liberia and Sierra Leone were digitized from the WHO situation report dated from 1 October 2014 (http://www.who.int/csr/disease/ebola/situation-reports/en/) (figure 3). To compare our predictions to those of previous reports [16], we also aggregated those data to form a regional epidemic curve for ‘West Africa’. In Guinea, this outbreak was taken to have started in the week ending 5 January 2014 and in Sierra Leone in that ending 8 June 2014. In Liberia, the outbreak was notified to WHO on 31 March 2014 (http://www.afro.who.int/en/clusters-a-programmes/dpc/epidemic-a-pandemic-alert-and-response/outbreak-news/4072-ebola-virus-disease-liberia.html), but few cases were reported until June; therefore, the week ending 1 June was deemed the start of the Liberian outbreak for simulation purposes. The data in figure 4 was downloaded from the repository maintained by C. M. Rivers (https://github.com/cmrivers/ebola) and ultimately derived from reports by the health ministries of the republics of Guinea, Sierra Leone and Liberia.

### (b) Model formulation

**Figure 6.**
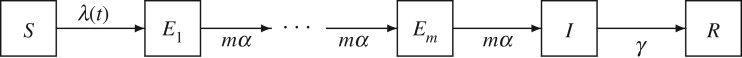
Schematic diagram of the transmission models used. *λ*(*t*) = *R*_0_*γI*(*t*)/*N* is the force of infection (i.e. the per-susceptible rate of infection). See §5(b) for explanation.

The models used were variants on the basic SEIR model (figure 6), using the method of stages to allow for a more realistic (Erlang) distribution of the incubation period [17,18]. The equations of the deterministic variant are
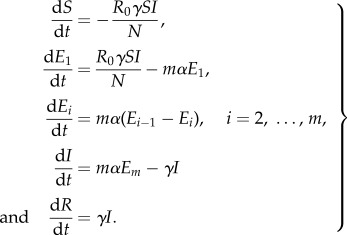

Here, *R*_0_ represents the basic reproduction number; 1/*α*, the average incubation period; *m*, the shape parameter for the incubation period distribution; 1/*γ*, the average infectious period; and *N*, the population size, assumed constant (electronic supplementary material, table B1). 

The stochastic variant was implemented as a continuous-time Markov process approximated via a multinomial modification of the *τ*-leap algorithm [14] with a fixed time step Δ*t* = 10^−2^ week.

To complete the model specification, we model the observation process. Let Δ*N_E_*_→*I*_(*t*_1_,*t*_2_) denote the total number of transitions from latent to infectious class (*E_m_* to *I*) occurring between times *t*_1_ and *t*_2_. Between times *t*−Δ*t* and *t*, where Δ*t* represents the reporting period, we write *H_t_* = Δ*N_E_*_→*I*_(*t* − Δ*t*,*t*) for the complete number of new infections during that time period. When we are fitting to cumulative case counts, we change the definition accordingly to *H_t_* = Δ*N_E_*_→*I*_(0,*t*). When using either type of data, we modelled the corresponding case report, *C_t_*, as a negative binomial: *C_t_* ∼ NegBin(*ρH_t_*,1/*k*). Thus, 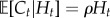 and 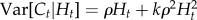, where *ρ* is the reporting probability and *k* the reporting overdispersion.

Descriptions of the methods used in the simulation study and in the model-based inferences drawn from actual data are given in the electronic supplementary material.

## Supplementary Material

Appendices
