# Supplementary material for: Avoidable errors in the modelling of outbreaks of emerging pathogens, with special reference to Ebola
Source: Proc Biol Sci. 2015 May 7;282(1806):20150347. doi: 10.1098/rspb.2015.0347 (PMC4426634; doi:10.1098/rspb.2015.0347)
Supplement: Appendices [file rspb20150347supp1.pdf]

Supplementary Material for:
Avoidable errors in the modeling of outbreaks of emerging pathogens, with special reference to Ebola

Aaron A. King[1,2,3,4,*]
Matthieu Domenech de Cellès[1]
Felicia M. G. Magpantay[1]
Pejman Rohani[1,2,4]

1 Department of Ecology & Evolutionary Biology, University of Michigan, Ann Arbor, Michigan, USA
2 Center for the Study of Complex Systems, University of Michigan, Ann Arbor, Michigan, USA
3 Department of Mathematics, University of Michigan, Ann Arbor, Michigan, USA
4 Fogarty International Center, National Institutes of Health, Bethesda, Maryland, USA
∗ E-mail: kingaa@umich.edu

Appendix A. Simulation study

To demonstrate the differences between fitting to raw incidence vs. cumulative incidence data, we performed a simulation study in which we fit the deterministic model variant to both types of data at three different levels of observation overdispersion: $k \in \{0, 0.2, 0.5\}$. For each overdispersion treatment, 500 simulated 39-week time series were generated from the stochastic model variant. The basic reproduction number was set to $R_0 = 1.4$; the incubation and infectious periods were fixed as in Table B1; the assumed population size was taken to be that of the Republic of Guinea. We assumed a reporting probability of $\rho = 0.2$ and that, at outbreak initiation, 10 individuals were infected. This set of parameter values yields a sample mean simulation visually comparable to the WHO data from Guinea, which display initally slow growth in the number of cases and later acceleration.

For each simulated data set, we estimated the basic reproduction number, R_0, the reporting probability, ρ, and the negative binomial overdispersion parameter, k. All other model parameters were fixed at their true values. Parameter estimation was accomplished using the trajectory matching algorithm (`traj.match`) from the R package `pomp` (King *et al.*, 2010). We constructed likelihood profiles over R_0 and, from these, obtained maximum likelihood point estimates and likelihood-ratio confidence intervals. The full process of obtaining likelihood profiles on model parameters by trajectory matching took approximately 1.2 hr on a 40-cpu cluster.

A second simulation study was performed, in which the deterministic variant of the model was fit to cumulative incidence data by ordinary least squares. This common procedure in effect assumes that measurement errors are independent and identically normally distributed. Results of this exercise are shown in Fig. A2 in a form comparable to that of Fig. 1. As in the results shown in the main text, confidence interval widths are erroneously under-estimated with the result that achieved coverage is far smaller than its nominal value.

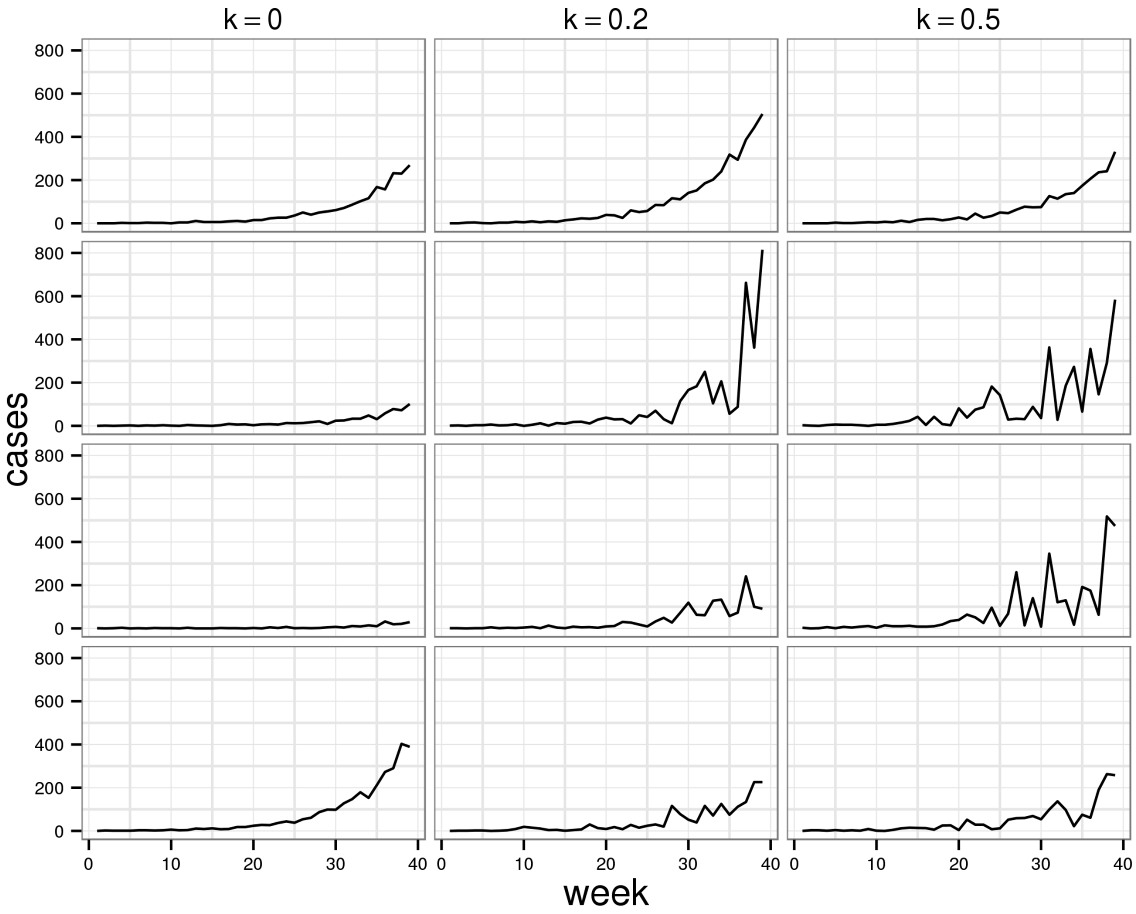

Figure A1: Twelve randomly-selected simulated datasets from the 1500 used in the simulation study. Four simulations are shown for each of three values of the negative binomial overdispersion parameter, k.

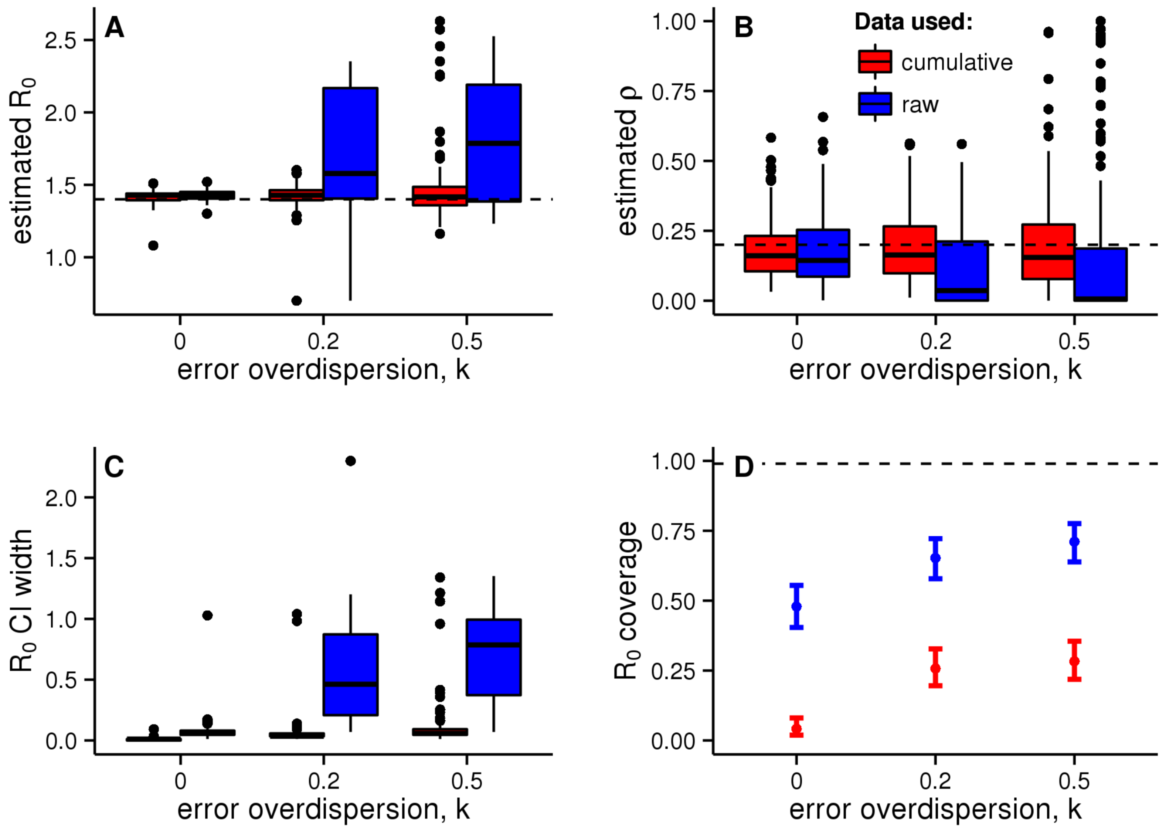

Figure A2: Results from simulation study fitting the deterministic model to cumulative incidence data using the method of least squares. The model was fit to both raw (blue) and accumulated (red) simulated incidence data. The same 1500 simulated data sets of length 39 wk used in Fig. 1 were used here. (A) Estimates of R_0. True value used in generating the data is shown by the dashed line. (B) Estimates of reporting probability, ρ. The dashed line shows the value used to generate the data. (C) Widths of nominal 99% profile likelihood confidence intervals (CI) for R_0. (D) Actual coverage of the CI, i.e., probability that the true value of R_0 lay within the CI. Ideally, actual coverage would agree with nominal coverage (99%, dashed line).

Appendix B. Model-based inference

Trajectory matching

Model parameters were initially estimated using trajectory matching. As in the simulation study, we initially fitted R_0, ρ, k and the initial conditions. However, profile likelihoods over ρ were flat, indicating a lack of identifiability in the reporting rate due to a trade-off between this parameter and initial conditions. Accordingly, we fixed $\rho = 0.2$. The flatness of the likelihood profiles indicates that this assumption has no effect on the quality of fit. All other model parameters were fixed at the known values given in Table B1.

Trajectory matching was used to compute likelihood profiles over R_0 and k. For each point in the profile, the other parameters and initial conditions were initialized at 40 points according to a latin hypersquare (Sobol') design. In all, the trajectory matching calculations required approximately 21 cpu hr of computation. Full details of the trajectory matching codes are provided in the Supplementary Material.

Table B1: Model parameters, with their interpretations, and their assumed values (parameters estimated from incidence data are so indicated) together with the source of evidence for the assumption.

Symbol	Meaning	Value	Citation
R_0	Basic reproduction number	Estimated	
$1/\alpha$	Average incubation period	11.4 da	WHO Ebola Response Team (2014)
m	Incubation period shape parameter	3	WHO Ebola Response Team (2014)
$1/\gamma$	Average infectious period	7 da	WHO Ebola Response Team (2014)
ρ	Reporting probability	0.2	Assumption
k	Reporting overdispersion	Estimated	
N	Population size	Guinea: 10.6M<br>Liberia: 4.1M<br>Sierra Leone: 6.2M	

Iterated filtering

Model parameters were estimated using the Iterated Filtering algorithm (IF2) (Ionides *et al.*, 2015), implemented as `mif` in the R package `pomp` (King *et al.*, 2010). For each country and each type of data, the parameter estimates along the trajectory-matching profiles were used to initialize the IF2 runs. From each initial point, we performed 60 IF2 iterations using 2×10^3 particles, hyperbolic cooling, and a random walk standard deviation (on the log scale) of 0.02 for all parameters and 1 for initial conditions. For the parameters estimated in each IF run, the log-likelihood was computed as the log of the mean likelihoods of 10 replicate filters, each with 5×10^3 particles. Approximate confidence intervals were then computed using the profile log-likelihood (Raue *et al.*, 2009). All details of these computations are provided in the Supplementary Material. Computing each of the profile likelihoods in Fig. 2 using iterated filtering took approximately 34 cpu hr of computation; all profile computations were accomplished in roughly 3.6 hr on a 100-cpu cluster.

Results

Table B2 shows the maximum likelihood parameter estimates (MLE). Fig. B1 shows a comparison of various summary statistics ("probes") computed both on the data and on model simulations.

To tease apart the consequences of failing to account for stochasticity from those of improperly fitting to cumulative data, we fit both deterministic and stochastic models each to both actual incidence and accumulated incidence data. It is important to recognize that the exercise of fitting the stochastic model to accumulated data is not something one would ever actually do. Indeed, at the outset incompatibility of model assumptions with the data becomes evident. To see this, let H_t be the true incidence (i.e., actual number of new infections) in reporting interval t and C_t be the number of reported cases in that interval. Because of measurement error, $C_t = H_t + \varepsilon_t$, where ε_t is the error. Let $h_t = \sum_{s=1}^{t} H_t$ and $c_t = \sum_{s=1}^{t} C_t$ be the accumulated true and reported incidence, respectively. Because $c_t = \sum_{s=1}^{t}(H_t + \varepsilon_t)$, the errors $c_t - h_t$ are not independent, which is the fundamental problem associated with fitting to cumulative incidence data, irrespective of whether the model for H_t is deterministic or stochastic. If one attempts to fit a stochastic model to c_t by modeling $c_t = h_t + \xi_t$, where ξ_t are measurement errors, one is confronted with the fact that, even though the accumulated data, c_t, and simulations of h_t are guaranteed to increase with time, simulations of c_t under this model will not in general be monotonically increasing.

Nevertheless, one naturally wonders about the relative importance of the choice to use a deterministic or stochastic model vs. using raw or accumulated incidence data. Although the answer will certainly depend on both model and data, and therefore vary from situation to situation, we present the comparison in the present case to partially satisfy this natural curiosity. For the SEIR model fit to the Sierra Leone outbreak data, Fig. B2 shows likelihood profiles for the four model-data combinations and Fig. B3 shows the corresponding forecasts.

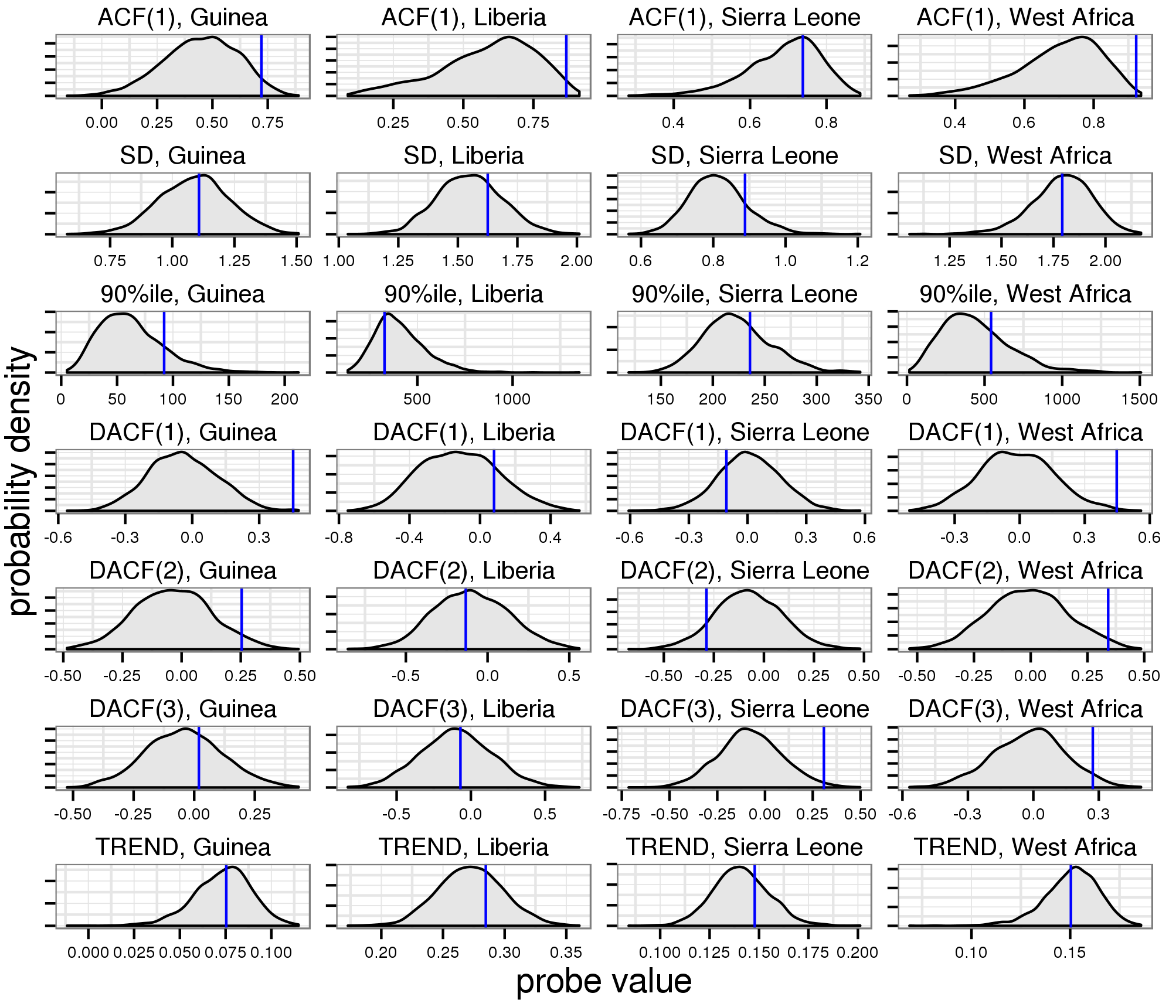

Figure B1: Additional summary statistics, or probes, computed on both stochastic model simulations and the data. In each panel, the probability density of the probes on the simulated data are shown in grey; the blue line indicates the value of the probe on the data. Probes include autocorrelation at lag 1 (ACF), standard deviation (SD) on log-transformed data, 90th percentile, the autocorrelation at lags 1, 2, and 3 wk after removing an exponential trend (DACF), and the exponential growth rate as obtained by log-linear regression (TREND).

Table B2: Parameter estimates for the stochastic and deterministic models on the raw data. MLE point estimates with nominal 95% confidence intervals are shown.

Parameter	R_0		k	
**Stochastic model, raw data**				
Guinea	1.2	(1.1–1.3)	0.37	(0.22–0.62)
Liberia	1.9	(1.7–2.2)	0.24	(0.12–0.52)
Sierra Leone	1.3	(1.2–1.4)	0.038	(0.02–0.1)
West Africa	1.5	(1.4–1.6)	0.17	(0.091–0.3)
**Deterministic model, raw data**				
Guinea	1.2	(1.2–1.3)	0.37	(0.23–0.63)
Liberia	1.9	(1.7–2.2)	0.23	(0.12–0.49)
Sierra Leone	1.3	(1.3–1.4)	0.04	(0.02–0.1)
West Africa	1.5	(1.4–1.5)	0.18	(0.11–0.32)
**Stochastic model, cumulative data**				
Guinea	1.2	(1.2–1.3)	0.01	(0.01–0.014)
Liberia	2	(1.9–2.1)	0.02	(0.01–0.051)
Sierra Leone	1.3	(1.2–1.3)	0.0005	(0–0.0022)
West Africa	1.5	(1.45–1.51)	0.01	(0.01–0.02)
**Deterministic model, cumulative data**				
Guinea	1.2	(1.2–1.24)	0.024	(0.02–0.051)
Liberia	2	(1.9–2.1)	0.02	(0.01–0.04)
Sierra Leone	1.3	(1.25–1.31)	0.0011	(0–0.002)
West Africa	1.5	(1.4–1.5)	0.02	(0.02–0.03)

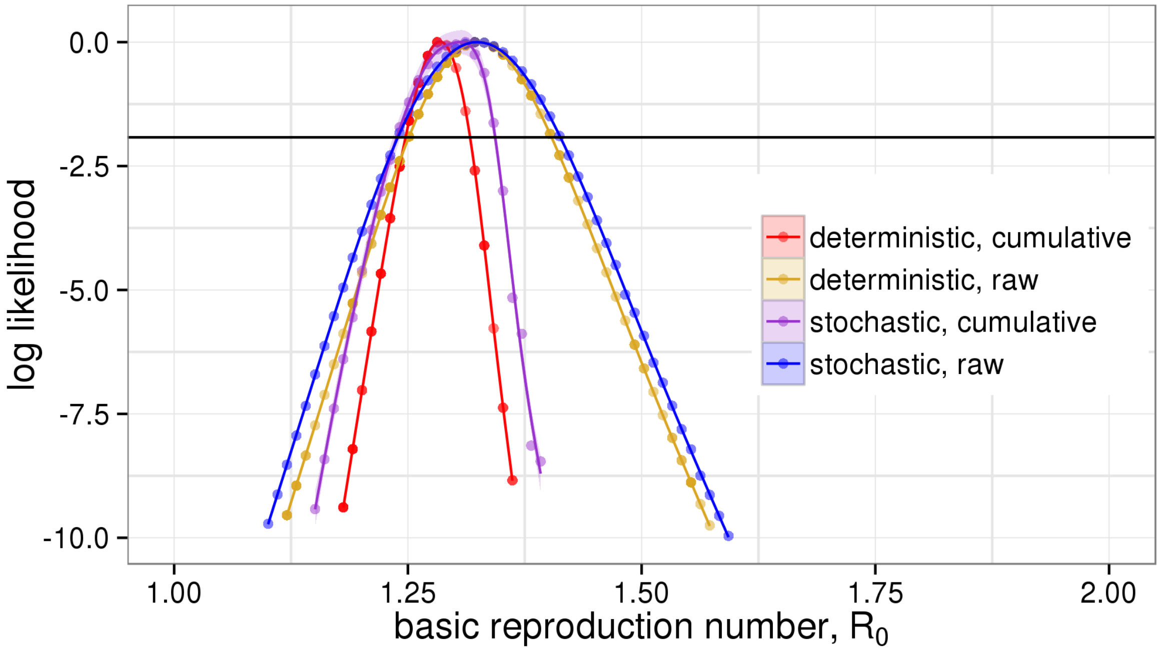

Figure B2: R_0 likelihood profiles for four model-data combinations for the SEIR model fit to the Sierra Leone outbreak.

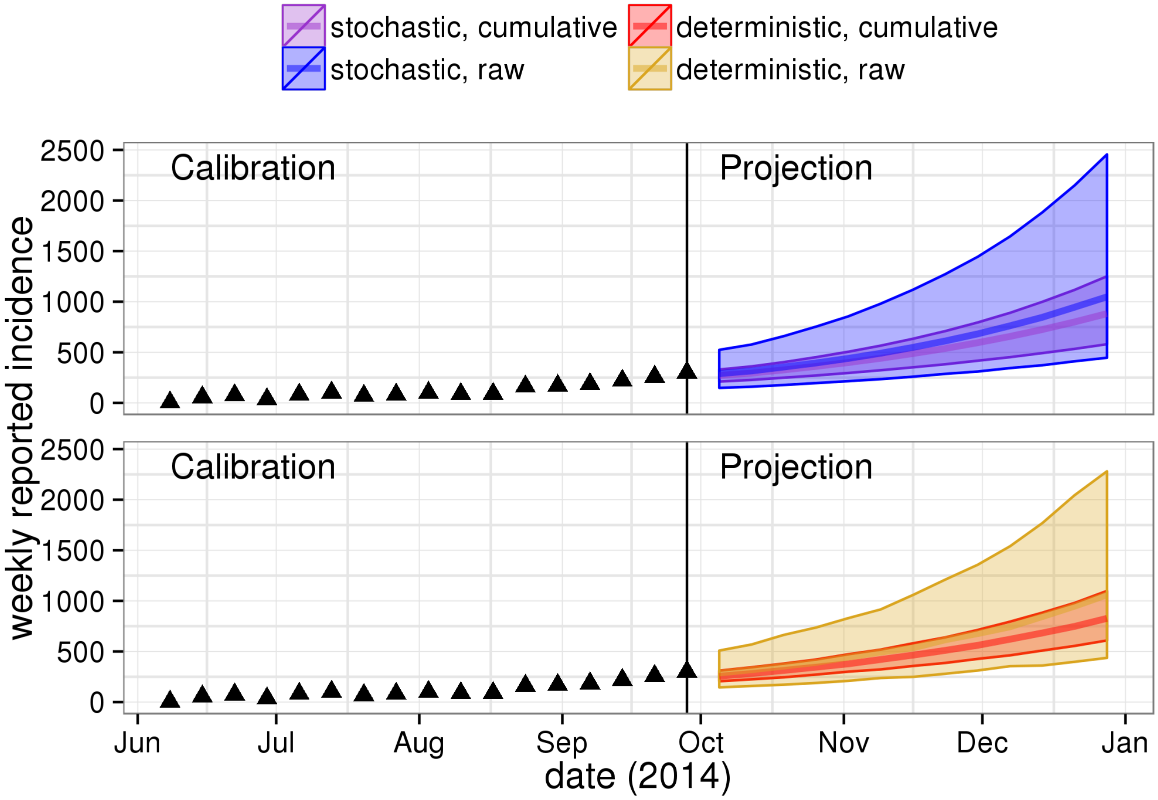

Figure B3: Forecast uncertainty for the Sierra Leone EBVD outbreak as a function of the model used and the data to which the model was fit. The ribbons show the median and 95% envelope of model simulations for the various models fit to raw and cumulative incidence data from the Sierra Leone outbreak. The data used in model fitting are shown using black triangles.

Appendix C. Data and codes

This appendix describes and displays the data and codes needed to fully reproduce all the results of the paper. All referenced files are provided on `datadryad.org`: `doi:10.5061/dryad.r5f30`.

Preliminaries

R packages

The following installs the necessary packages, if they are not already installed.

```
pkgs <- c("plyr","pomp","reshape2","magrittr","ggplot2","scales",
          "foreach","doMC","doMPI","iterators")
```

```
ipkgs <- rownames(installed.packages())
npkgs <- setdiff(pkgs,ipkgs)
if (length(npkgs)>0) install.packages(npkgs)
if (packageVersion("pomp")<"0.62-5") {
  install.packages("pomp", repos="http://R-Forge.R-project.org")
  }
```

The following are the version numbers of these packages used in performing the study.

```
R.version.string
sapply(pkgs,function(x)as.character(packageVersion(x)))
```

```
## [1] "R version 3.1.2 (2014-10-31)"
##      plyr      pomp  reshape2  magrittr   ggplot2    scales   foreach
##   "1.8.1"  "0.62.5"   "1.4.1"     "1.5"   "1.0.0"   "0.2.4"   "1.4.2"
##      doMC     doMPI iterators
##   "1.3.3"   "0.2.1"   "1.0.7"
```

MPI

Most of the codes described below are designed to be run on a cluster using OpenMPI. It will be assumed that an MPI hostfile, `hosts`, exists, which specifes the hosts to be used. An example `hosts` file has contents

```
localhost slots=1 max-slots=1
node1 slots=0 max-slots=10
node2 slots=0 max-slots=10
node3 slots=0 max-slots=10
node4 slots=0 max-slots=10
```

Here, `node1`, `node2`, `node3`, and `node4` are the compute nodes in this small cluster. Each has at least 10 cores.

Model implementation

The file `ebola.R`, shown below, will be sourced by the each of the codes below. It defines a function `ebolaModel` that constructs a `pomp` object holding the Ebola data and the model codes.

Contents of the file `ebola.R`:

```
require(pomp)
require(plyr)
require(reshape2)
require(magrittr)

stopifnot(packageVersion("pomp")>="0.62-4")

WHO.situation.report.Oct.1 <- '
week,Guinea,Liberia,SierraLeone
1,2.244,,
2,2.244,,
3,0.073,,
4,5.717,,
5,3.954,,
6,5.444,,
7,3.274,,
8,5.762,,
9,7.615,,
10,7.615,,
11,27.392,,
12,17.387,,
13,27.115,,
14,29.29,,
15,27.84,,
16,16.345,,
17,10.917,,
18,11.959,,
19,11.959,,
20,8.657,,
21,26.537,,
22,47.764,3.517,
23,26.582,1.043,5.494
24,32.967,18,57.048
25,18.707,16.34,76.022
26,24.322,13.742,36.768
27,4.719,10.155,81.929
28,7.081,24.856,102.632
29,8.527,53.294,69.823
30,92.227,70.146,81.783
31,26.423,139.269,99.775
32,16.549,65.66,88.17
33,36.819,240.645,90.489
34,92.08,274.826,161.54
35,101.03,215.56,168.966
36,102.113,388.553,186.144
37,83.016,410.299,220.442
38,106.674,300.989,258.693
39,55.522,240.237,299.546
'

## Population sizes in Guinea, Liberia, and Sierra Leone (census 2014)
populations <- c(Guinea=10628972,Liberia=4092310,SierraLeone=6190280)
populations["WestAfrica"] <- sum(populations)

read.csv(text=WHO.situation.report.Oct.1,stringsAsFactors=FALSE) %>%
  mutate(date=seq(from=as.Date("2014-01-05"),length=length(week),by='week')) %>%
  melt(id=c("week","date"),variable.name="country",value.name="cases") %>%
  mutate(deaths=NA) -> dat

## Parameter transformations

paruntrans <- Csnippet('
  double *IC = &S_0;
  double *TIC = &TS_0;
```

```
  TR0 = log(R0);
  Trho = logit(rho);
  Tk = log(k);
  to_log_barycentric(TIC,IC,4);
')

partrans <- Csnippet('
  double *IC = &S_0;
  double *TIC = &TS_0;
  TR0 = exp(R0);
  Trho = expit(rho);
  Tk = exp(k);
  from_log_barycentric(TIC,IC,4);
')

##  Measurement model: hierarchical model for cases
## p(C_t | H_t): Negative binomial with mean rho*H_t and variance rho*H_t*(1+k*rho*H_t)
dObs <- Csnippet('
  double f;
  if (k > 0.0)
    f = dnbinom_mu(nearbyint(cases),1.0/k,rho*N_EI,1);
  else
    f = dpois(nearbyint(cases),rho*N_EI,1);
  lik = (give_log) ? f : exp(f);
')

rObs <- Csnippet('
  if (k > 0) {
    cases = rnbinom_mu(1.0/k,rho*N_EI);
    deaths = rnbinom_mu(1.0/k,rho*cfr*N_IR);
  } else {
    cases = rpois(rho*N_EI);
    deaths = rpois(rho*cfr*N_IR);
  }')

### measurement model for ordinary least-squares
dObsLS <- Csnippet('
  double f;
  f = dnorm(cases,rho*N_EI,k,1);
  lik = (give_log) ? f : exp(f);
')

rObsLS <- Csnippet('
  cases = rnorm(rho*N_EI,k);
  deaths = NA_REAL;
')

## Process model simulator
rSim <- Csnippet('
  double lambda, beta;
  double *E = &E1;
  beta = R0 * gamma; // Transmission rate
  lambda = beta * I / N; // Force of infection
  int i;

  // Transitions
  // From class S
  double transS = rbinom(S, 1.0 - exp(- lambda * dt)); // No of infections
  // From class E
  double transE[nstageE]; // No of transitions between classes E
  for(i = 0; i < nstageE; i++){
    transE[i] = rbinom(E[i], 1.0 - exp(- nstageE * alpha * dt));
  }
  // From class I
  double transI = rbinom(I, 1.0 - exp(- gamma * dt)); // No of transitions I->R

  // Balance the equations
  S -= transS;
  E[0] += transS - transE[0];
  for(i=1; i < nstageE; i++) {
    E[i] += transE[i-1] - transE[i];
  }
  I += transE[nstageE - 1] - transI;
```

```
  R += transI;
  N_EI += transE[nstageE - 1]; // No of transitions from E to I
  N_IR += transI; // No of transitions from I to R
')

## Deterministic skeleton (an ODE), used in trajectory matching
skel <- Csnippet('
  double lambda, beta;
  double *E = &E1;
  double *DE = &DE1;
  beta = R0 * gamma; // Transmission rate
  lambda = beta * I / N; // Force of infection
  int i;

  // Balance the equations
  DS = - lambda * S;
  DE[0] = lambda * S - nstageE * alpha * E[0];
  for (i=1; i < nstageE; i++)
    DE[i] = nstageE * alpha * (E[i-1]-E[i]);
  DI = nstageE * alpha * E[nstageE-1] - gamma * I;
  DR = gamma * I;
  DN_EI = nstageE * alpha * E[nstageE-1];
  DN_IR = gamma * I;
')

ebolaModel <- function (country=c("Guinea", "SierraLeone", "Liberia", "WestAfrica"),
                        data = NULL,
                        timestep = 0.01, nstageE = 3L,
                        type = c("raw","cum"), na.rm = FALSE, least.sq = FALSE) {

  type <- match.arg(type)
  ctry <- match.arg(country)
  pop <- unname(populations[ctry])

  ## Incubation period is supposed to be Gamma distributed with shape parameter 3
  ## and mean 11.4 days.  The discrete-time formula is used to calculate the
  ## corresponding alpha (cf He et al., Interface 2010).
  ## Case-fatality ratio is fixed at 0.7 (cf WHO Ebola response team, NEJM 2014)
  incubation_period <- 11.4/7
  infectious_period <- 7/7
  index_case <- 10/pop
  dt <- timestep
  nstageE <- as.integer(nstageE)

  globs <- paste0("static int nstageE = ",nstageE,";");

  theta <- c(N=pop,R0=1.4,
             alpha=-1/(nstageE*dt)*log(1-nstageE*dt/incubation_period),
             gamma=-log(1-dt/infectious_period)/dt,
             rho=0.2,cfr=0.7,
             k=0,
             S_0=1-index_case,E_0=index_case/2-5e-9,
             I_0=index_case/2-5e-9,R_0=1e-8)

  if (is.null(data)) {
    if (ctry=="WestAfrica") {
      dat <- ddply(dat,~week,summarize,
                   cases=sum(cases,na.rm=TRUE),
                   deaths=sum(deaths,na.rm=TRUE))
      } else {
        dat <- subset(dat,country==ctry,select=-country)
        }
    } else {
      dat <- data
      }

  if (na.rm) {
    dat <- mutate(subset(dat,!is.na(cases)),week=week-min(week)+1)
    }
  if (type=="cum") {
    dat <- mutate(dat,cases=cumsum(cases),deaths=cumsum(deaths))
    }
```

```
  ## Create the pomp object
  pomp(
    data=dat[c("week","cases","deaths")],
    times="week",
    t0=0,
    params=theta,
    globals=globs,
    obsnames=c("cases","deaths"),
    statenames=c("S","E1","I","R","N_EI","N_IR"),
    zeronames=if (type=="raw") c("N_EI","N_IR") else character(0),
    paramnames=c("N","R0","alpha","gamma","rho","k","cfr",
                 "S_0","E_0","I_0","R_0"),
    nstageE=nstageE,
    dmeasure=if (least.sq) dObsLS else dObs,
    rmeasure=if (least.sq) rObsLS else rObs,
    rprocess=discrete.time.sim(step.fun=rSim,delta.t=timestep),
    skeleton=skel,
    skeleton.type="vectorfield",
    parameter.transform=partrans,
    parameter.inv.transform=paruntrans,
    initializer=function (params, t0, nstageE, ...) {
      all.state.names <- c("S",paste0("E",1:nstageE),"I","R","N_EI","N_IR")
      comp.names <- c("S",paste0("E",1:nstageE),"I","R")
      x0 <- setNames(numeric(length(all.state.names)),all.state.names)
      frac <- c(params["S_0"],rep(params["E_0"]/nstageE,nstageE),params["I_0"],params["R_0"])
      x0[comp.names] <- round(params["N"]*frac/sum(frac))
      x0
      }
    ) -> po
  }

c("ebolaModel")
```

Simulation study

The codes in file, `simstudy.R`, shown below, perform the simulation study computations. Note that it assumes that file `ebola.R` (contents displayed above) is present in the working directory. On a cluster with `R` and `OpenMPI` installed, in a directory containing `simstudy.R`, `ebola.R`, and the hostfile `hosts`, execute the computations with a command such as:

```
mpirun -hostfile hosts -np 41 Rscript --vanilla simstudy.R
```

Contents of file `simstudy.R`:

```
require(pomp)
require(plyr)
require(reshape2)
require(magrittr)
options(stringsAsFactors=FALSE)

require(foreach)
require(doMPI)
require(iterators)

source("ebola.R")
noexport <- c("ebolaModel")

cl <- startMPIcluster()
registerDoMPI(cl)

bake <- function (file, expr) {
  if (file.exists(file)) {
    readRDS(file)
```

```
    } else {
      val <- eval(expr)
      saveRDS(val,file=file)
      val
      }
  }

## trajectory matching simulation study

tic <- Sys.time()

po <- ebolaModel(country="Guinea",timestep=0.1)
params <- parmat(coef(po),3)
params["k",] <- c(0,0.2,0.5)
paramnames <- names(coef(po))

nsims <- 500

bake(file="sims.rds",{
  simulate(po,params=params,nsim=nsims,seed=208335746L,
           as.data.frame=TRUE,obs=TRUE) %>%
    rename(c(time="week")) %>%
    mutate(k=params["k",((as.integer(sim)-1)%%ncol(params))+1])
  }) -> simdat

pompUnload(po)

bake(file="tm-sim-profiles-R0.rds",{
  foreach(simul=1:nsims,
          .combine=rbind,.inorder=FALSE,
          .noexport=noexport,
          .options.mpi=list(chunkSize=10,seed=1598260027L,info=TRUE)) %:%
    foreach(type=c("raw","cum"),.combine=rbind,.inorder=FALSE) %dopar%
    {
      dat <- subset(simdat,sim==simul,select=c(week,cases,deaths))
      tm <- ebolaModel(country="Guinea",data=dat,type=as.character(type))
      st <- params[,(simul-1)%%3+1]
      true.k <- unname(st["k"])
      true.R0 <- unname(st["R0"])
      true.rho <- unname(st["rho"])
      st["k"] <- st["k"]+1e-6
      tm <- traj.match(tm,start=st,est=c("R0","k","rho"),transform=TRUE)
      if (coef(tm,"rho")==1) coef(tm,"rho") <- 0.999
      if (coef(tm,"rho")==0) coef(tm,"rho") <- 0.001
      tm <- traj.match(tm,est=c("R0","k","rho"),method='subplex',transform=TRUE)
      pompUnload(tm)
      data.frame(sim=simul,type=as.character(type),
                 true.k=true.k,true.R0=true.R0,true.rho=true.rho,
                 as.list(coef(tm)),loglik=logLik(tm),
                 conv=tm$convergence)
      } -> fits

  foreach (fit=iter(fits,by="row"),
           .noexport=noexport,
           .combine=rbind,.inorder=FALSE) %:%
    foreach (r0=seq(from=0.7,to=3,length=200),
             .combine=rbind,.inorder=FALSE,
             .options.mpi=list(chunkSize=200,seed=1598260027L,info=TRUE)) %dopar%
    {
      dat <- subset(simdat,sim==fit$sim,select=c(week,cases,deaths))
      tm <- ebolaModel(country="Guinea",data=dat,type=as.character(fit$type))
      coef(tm) <- unlist(fit[paramnames])
      coef(tm,"R0") <- r0
      if (coef(tm,"rho")==1) coef(tm,"rho") <- 0.999
      if (coef(tm,"rho")==0) coef(tm,"rho") <- 0.001
      tm <- traj.match(tm,est=c("k","rho"),transform=TRUE)
      if (coef(tm,"rho")==1) coef(tm,"rho") <- 0.999
      if (coef(tm,"rho")==0) coef(tm,"rho") <- 0.001
      tm <- traj.match(tm,est=c("k","rho"),transform=TRUE,method='subplex')
      pompUnload(tm)
      data.frame(sim=fit$sim,type=fit$type,
                 true.k=fit$true.k,true.R0=fit$true.R0,true.rho=fit$true.rho,
                 as.list(coef(tm)),loglik=logLik(tm),
```

```
                 conv=tm$convergence)
      }
  }) -> profiles

bake(file="tm-sim-fits.rds",{

  ddply(profiles,~type+sim+true.k,subset,loglik==max(loglik)) -> starts

  foreach(fit=iter(starts,by="row"),.combine=rbind,.inorder=FALSE,
          .noexport=noexport,
          .options.mpi=list(chunkSize=30,seed=1598260027L,info=TRUE)) %dopar%
    {
      dat <- subset(simdat,sim==fit$sim,select=c(week,cases,deaths))
      tm <- ebolaModel(country="Guinea",data=dat,type=as.character(fit$type))
      coef(tm) <- unlist(fit[paramnames])
      if (coef(tm,"rho")==1) coef(tm,"rho") <- 0.999
      if (coef(tm,"rho")==0) coef(tm,"rho") <- 0.001
      tm <- traj.match(tm,est=c("k","rho"),transform=TRUE)
      if (coef(tm,"rho")==1) coef(tm,"rho") <- 0.999
      if (coef(tm,"rho")==0) coef(tm,"rho") <- 0.001
      tm <- traj.match(tm,est=c("k","rho"),transform=TRUE,method='subplex')
      pompUnload(tm)
      data.frame(sim=fit$sim,type=fit$type,
                 true.k=fit$true.k,true.R0=fit$true.R0,true.rho=fit$true.rho,
                 as.list(coef(tm)),loglik=logLik(tm),
                 conv=tm$convergence)
      }
  }) -> fits

toc <- Sys.time()
print(toc-tic)

## trajectory matching with least squares simulation study

tic <- Sys.time()

bake(file="ls-sim-profiles-R0.rds",{

  foreach(simul=1:nsims,
          .combine=rbind,.inorder=FALSE,
          .noexport=noexport,
          .options.mpi=list(chunkSize=10,seed=1598260027L,info=TRUE)) %:%
    foreach(type=c("raw","cum"),.combine=rbind,.inorder=FALSE) %dopar%
    {
      dat <- subset(simdat,sim==simul,select=c(week,cases,deaths))
      tm <- ebolaModel(country="Guinea",data=dat,type=as.character(type),
                       least.sq=TRUE)
      st <- params[,(simul-1)%%3+1]
      true.k <- unname(st["k"])
      true.R0 <- unname(st["R0"])
      true.rho <- unname(st["rho"])
      st["k"] <- 10
      tm <- traj.match(tm,start=st,est=c("R0","k","rho"),transform=TRUE)
      if (coef(tm,"rho")==1) coef(tm,"rho") <- 0.999
      if (coef(tm,"rho")==0) coef(tm,"rho") <- 0.001
      tm <- traj.match(tm,est=c("R0","k","rho"),method='subplex',transform=TRUE)
      pompUnload(tm)
      data.frame(sim=simul,type=as.character(type),
                 true.k=true.k,true.R0=true.R0,true.rho=true.rho,
                 as.list(coef(tm)),loglik=logLik(tm),
                 conv=tm$convergence)
      } -> fits

  foreach (fit=iter(fits,by="row"),
           .noexport=noexport,
           .combine=rbind,.inorder=FALSE) %:%
    foreach (r0=seq(from=0.7,to=3,length=200),
             .combine=rbind,.inorder=FALSE,
             .options.mpi=list(chunkSize=200,seed=1598260027L,info=TRUE)) %dopar%
    {
      dat <- subset(simdat,sim==fit$sim,select=c(week,cases,deaths))
      tm <- ebolaModel(country="Guinea",data=dat,type=as.character(fit$type),
                       least.sq=TRUE)
```

```
      coef(tm) <- unlist(fit[paramnames])
      coef(tm,"R0") <- r0
      if (coef(tm,"rho")==1) coef(tm,"rho") <- 0.999
      if (coef(tm,"rho")==0) coef(tm,"rho") <- 0.001
      tm <- traj.match(tm,est=c("k","rho"),transform=TRUE)
      if (coef(tm,"rho")==1) coef(tm,"rho") <- 0.999
      if (coef(tm,"rho")==0) coef(tm,"rho") <- 0.001
      tm <- traj.match(tm,est=c("k","rho"),transform=TRUE,method='subplex')
      pompUnload(tm)
      data.frame(sim=fit$sim,type=fit$type,
                 true.k=fit$true.k,true.R0=fit$true.R0,true.rho=fit$true.rho,
                 as.list(coef(tm)),loglik=logLik(tm),
                 conv=tm$convergence)
      }
  }) -> profiles

bake(file="ls-sim-fits.rds",{

  ddply(profiles,~type+sim+true.k,subset,loglik==max(loglik)) -> starts

  foreach(fit=iter(starts,by="row"),.combine=rbind,.inorder=FALSE,
          .noexport=noexport,
          .options.mpi=list(chunkSize=30,seed=1598260027L,info=TRUE)) %dopar%
    {
      dat <- subset(simdat,sim==fit$sim,select=c(week,cases,deaths))
      tm <- ebolaModel(country="Guinea",data=dat,type=as.character(fit$type),
                       least.sq=TRUE)
      coef(tm) <- unlist(fit[paramnames])
      if (coef(tm,"rho")==1) coef(tm,"rho") <- 0.999
      if (coef(tm,"rho")==0) coef(tm,"rho") <- 0.001
      tm <- traj.match(tm,est=c("k","rho"),transform=TRUE)
      if (coef(tm,"rho")==1) coef(tm,"rho") <- 0.999
      if (coef(tm,"rho")==0) coef(tm,"rho") <- 0.001
      tm <- traj.match(tm,est=c("k","rho"),transform=TRUE,method='subplex')
      pompUnload(tm)
      data.frame(sim=fit$sim,type=fit$type,
                 true.k=fit$true.k,true.R0=fit$true.R0,true.rho=fit$true.rho,
                 as.list(coef(tm)),loglik=logLik(tm),
                 conv=tm$convergence)
      }
  }) -> fits

toc <- Sys.time()
print(toc-tic)

closeCluster(cl)
mpi.quit()
```

Parameter estimation

The codes in file `profiles.R` use trajectory matching and iterated filtering to estimate model parameters for all four countries and both types of data. Again, this is designed to be run on an MPI cluster. In a directory with the files `hosts` and `ebola.R` (see above), execute these computations via a command like

```
mpirun -hostfile hosts -np 101 Rscript --vanilla profiles.R
```

Contents of file `profiles.R`:

```
require(pomp)
require(plyr)
require(reshape2)
require(magrittr)
options(stringsAsFactors=FALSE)
```

```
require(foreach)
require(doMPI)
require(iterators)

source("ebola.R")

foreach (country=c("SierraLeone","Liberia","Guinea","WestAfrica"),
         .inorder=TRUE,.combine=c) %:%
  foreach (type=c("raw","cum"),.inorder=TRUE,.combine=c) %do%
  {
    ebolaModel(country=country,type=type,na.rm=TRUE)
    } -> models
dim(models) <- c(2,4)
dimnames(models) <- list(c("raw","cum"),
                         c("SierraLeone","Liberia","Guinea","WestAfrica"))

noexport <- c("models")

cl <- startMPIcluster()
registerDoMPI(cl)

bake <- function (file, expr) {
  if (file.exists(file)) {
    readRDS(file)
    } else {
      val <- eval(expr)
      saveRDS(val,file=file)
      val
      }
  }

## trajectory matching: R0 profile

bake(file="tm-fits-R0.rds",{

  starts <- profileDesign(R0=seq(1,3,length=200),
                          upper=c(k=1),
                          lower=c(k=1e-8),
                          nprof=40)

  foreach (start=iter(starts,by='row'),
           .combine=rbind,.inorder=FALSE,
           .noexport=noexport,
           .options.mpi=list(chunkSize=100,seed=2016138277L,info=TRUE)
           ) %:%
    foreach (type=c("raw","cum"),.combine=rbind,.inorder=FALSE) %:%
    foreach (country=c("SierraLeone","Liberia","Guinea","WestAfrica"),
             .combine=rbind,.inorder=FALSE) %dopar%
    {
      tm <- models[type,country][[1]]
      tic <- Sys.time()
      coef(tm,names(start)) <- unname(unlist(start))
      coef(tm,"rho") <- 0.2
      tm <- traj.match(tm,est=c("k","E_0","I_0"),transform=TRUE)
      if (coef(tm,"k")==0) coef(tm,"k") <- 1e-8
      if (coef(tm,"E_0")==0) coef(tm,"E_0") <- 1e-12
      if (coef(tm,"I_0")==0) coef(tm,"I_0") <- 1e-12
      tm <- traj.match(tm,method='subplex',control=list(maxit=1e5))
      toc <- Sys.time()
      etime <- toc-tic
      units(etime) <- "hours"
      data.frame(country=country,type=type,as.list(coef(tm)),
                 loglik=logLik(tm),conv=tm$convergence,
                 etime=as.numeric(etime))
      } %>% mutate(sum=S_0+E_0+I_0+R_0,
                   S_0=round(N*S_0/sum),
                   E_0=round(N*E_0/sum),
                   I_0=round(N*I_0/sum),
                   R_0=round(N*R_0/sum)) %>%
    subset(conv %in% c(0,1),select=-sum) %>%
    unique()

  }) -> profR0
```

```r
## trajectory matching: k profile

bake(file="tm-fits-k.rds",{

  starts <- profileDesign(k=seq(0,1,length=100),
                          upper=c(R0=1),
                          lower=c(R0=3),
                          nprof=40)

  foreach (start=iter(starts,by='row'),
           .combine=rbind,.inorder=FALSE,
           .noexport=noexport,
           .options.mpi=list(chunkSize=100,seed=2016138277L,info=TRUE)
           ) %:%
    foreach (type=c("raw","cum"),.combine=rbind,.inorder=FALSE) %:%
    foreach (country=c("SierraLeone","Liberia","Guinea","WestAfrica"),
             .combine=rbind,.inorder=FALSE) %dopar%
    {
      tm <- models[type,country][[1]]
      tic <- Sys.time()
      coef(tm,names(start)) <- unname(unlist(start))
      coef(tm,"rho") <- 0.2
      tm <- traj.match(tm,est=c("R0","E_0","I_0"),transform=TRUE)
      if (coef(tm,"E_0")==0) coef(tm,"E_0") <- 1e-12
      if (coef(tm,"I_0")==0) coef(tm,"I_0") <- 1e-12
      tm <- traj.match(tm,method='subplex',control=list(maxit=1e5))
      toc <- Sys.time()
      etime <- toc-tic
      units(etime) <- "hours"
      data.frame(country=country,type=type,as.list(coef(tm)),
                 loglik=logLik(tm),conv=tm$convergence,
                 etime=as.numeric(etime))
    } %>% mutate(sum=S_0+E_0+I_0+R_0,
                 S_0=round(N*S_0/sum),
                 E_0=round(N*E_0/sum),
                 I_0=round(N*I_0/sum),
                 R_0=round(N*R_0/sum)) %>%
    subset(conv %in% c(0,1),select=-sum) %>%
    unique()

  }) -> profk

## All trajectory matching computations
ldply(list(R0=profR0,k=profk),.id='profile') -> profTM

## Iterated filtering, R0 profile

bake(file="if-fits-R0_a.rds",{

  profTM %>% subset(profile=="R0") %>%
    ddply(~country+type,subset,
          is.finite(loglik)&loglik>max(loglik)-20) %>%
    ddply(~country+type+R0,subset,
          loglik==max(loglik),
          select=-c(loglik,etime,conv,profile)) -> pars

  foreach (start=iter(pars,by='row'),
           .combine=rbind,.inorder=FALSE,
           .options.mpi=list(chunkSize=1,seed=1264624821L),
           .noexport=noexport) %dopar%
    {
      tic <- Sys.time()

      country <- as.character(start$country)
      type <- as.character(start$type)
      st <- unlist(subset(start,select=-c(country,type)))

      po <- models[type,country][[1]]
      coef(po,names(st)) <- st
      if (coef(po,"E_0")==0) coef(po,"E_0") <- 1e-5
      if (coef(po,"I_0")==0) coef(po,"I_0") <- 1e-5

      mf <- mif(po, Nmif=10,
```

```r
                rw.sd = c(k=0.02,E_0=1,I_0=1),
                ivps = c("E_0","I_0"),
                Np = 2000,
                var.factor = 2,
                method = "mif2",
                cooling.type = "hyperbolic",
                cooling.fraction = 0.5,
                transform = TRUE,
                verbose = FALSE)
      mf <- continue(mf, Nmif = 50, cooling.fraction = 0.1)

      ## Runs 10 particle filters to assess Monte Carlo error in likelihood
      pf <- replicate(10,pfilter(mf,Np=5000,max.fail=Inf))
      ll <- sapply(pf,logLik)
      ll <- logmeanexp(ll, se = TRUE)
      nfail <- sapply(pf,getElement,"nfail")
      toc <- Sys.time()
      etime <- toc-tic
      units(etime) <- "hours"

      data.frame(country=country,type=type,as.list(coef(mf)),
                 loglik = ll[1],
                 loglik.se = ll[2],
                 nfail.min = min(nfail),
                 nfail.max = max(nfail),
                 etime = as.numeric(etime))
      }
  }) -> profR0

## Filter once more on maxima

bake(file="if-fits-R0.rds",{

  profR0 %>% subset(is.finite(loglik)&nfail.max==0) %>%
    ddply(~country+type+R0,subset,rank(-loglik)<=5) %>%
    subset(select=-c(loglik,loglik.se,nfail.max,nfail.min,etime)) -> pars

  foreach (start=iter(pars,by='row'),
           .combine=rbind,.inorder=FALSE,
           .options.mpi=list(chunkSize=1,seed=1264624821L),
           .noexport=noexport) %dopar%
    {
      tic <- Sys.time()

      country <- as.character(start$country)
      type <- as.character(start$type)
      st <- unlist(subset(start,select=-c(country,type)))

      po <- models[type,country][[1]]
      coef(po,names(st)) <- unname(st)

      ## Runs 10 particle filters to assess Monte Carlo error in likelihood
      pf <- try(replicate(10,pfilter(po,Np=5000,max.fail=Inf)))

      toc <- Sys.time()
      etime <- toc-tic
      units(etime) <- "hours"

      ll <- sapply(pf,logLik)
      ll <- logmeanexp(ll, se = TRUE)
      nfail <- sapply(pf,getElement,"nfail")

      data.frame(country=country,type=type,as.list(coef(po)),
                 loglik = ll[1],
                 loglik.se = ll[2],
                 nfail.min = min(nfail),
                 nfail.max = max(nfail),
                 etime = as.numeric(etime))
      }
  }) -> profR0

## Iterated filtering, k profile
```

```r
bake(file="if-fits-k_a.rds",{

  profTM %>% subset(profile=="k") %>%
    ddply(~country+type,subset,
          is.finite(loglik)&loglik>max(loglik)-20) %>%
    ddply(~country+type+k,subset,
          loglik==max(loglik),
          select=-c(loglik,etime,conv,profile)) -> pars

  foreach (start=iter(pars,by='row'),
           .combine=rbind,.inorder=FALSE,
           .options.mpi=list(chunkSize=1,seed=1264624821L),
           .noexport=noexport) %dopar%
    {
      tic <- Sys.time()

      country <- as.character(start$country)
      type <- as.character(start$type)
      st <- unlist(subset(start,select=-c(country,type)))

      po <- models[type,country][[1]]
      coef(po,names(st)) <- st
      if (coef(po,"E_0")==0) coef(po,"E_0") <- 1e-5
      if (coef(po,"I_0")==0) coef(po,"I_0") <- 1e-5

      mf <- mif(po, Nmif=10,
                rw.sd = c(R0=0.02,E_0=1,I_0=1),
                ivps = c("E_0","I_0"),
                Np = 2000,
                var.factor = 2,
                method = "mif2",
                cooling.type = "hyperbolic",
                cooling.fraction = 0.5,
                transform = TRUE,
                verbose = FALSE)
      mf <- continue(mf, Nmif = 50, cooling.fraction = 0.1)

      ## Runs 10 particle filters to assess Monte Carlo error in likelihood
      pf <- replicate(10,pfilter(mf,Np=5000,max.fail=Inf))
      ll <- sapply(pf,logLik)
      ll <- logmeanexp(ll, se = TRUE)
      nfail <- sapply(pf,getElement,"nfail")
      toc <- Sys.time()
      etime <- toc-tic
      units(etime) <- "hours"

      data.frame(country=country,type=type,as.list(coef(mf)),
                 loglik = ll[1],
                 loglik.se = ll[2],
                 nfail.min = min(nfail),
                 nfail.max = max(nfail),
                 etime = as.numeric(etime))
    }
}) -> profk

## Filter once more on maxima

bake(file="if-fits-k.rds",{

  profk %>% subset(is.finite(loglik)&nfail.max==0) %>%
    ddply(~country+type+R0,subset,rank(-loglik)<=5) %>%
    subset(select=-c(loglik,loglik.se,nfail.max,nfail.min,etime)) -> pars

  foreach (start=iter(pars,by='row'),
           .combine=rbind,.inorder=FALSE,
           .options.mpi=list(chunkSize=1,seed=1264624821L),
           .noexport=noexport) %dopar%
    {
      tic <- Sys.time()

      country <- as.character(start$country)
      type <- as.character(start$type)
      st <- unlist(subset(start,select=-c(country,type)))
```

```
    po <- models[type,country][[1]]
    coef(po,names(st)) <- unname(st)

    ## Runs 10 particle filters to assess Monte Carlo error in likelihood
    pf <- try(replicate(10,pfilter(po,Np=5000,max.fail=Inf)))

    toc <- Sys.time()
    etime <- toc-tic
    units(etime) <- "hours"

    ll <- sapply(pf,logLik)
    ll <- logmeanexp(ll, se = TRUE)
    nfail <- sapply(pf,getElement,"nfail")

    data.frame(country=country,type=type,as.list(coef(po)),
               loglik = ll[1],
               loglik.se = ll[2],
               nfail.min = min(nfail),
               nfail.max = max(nfail),
               etime = as.numeric(etime))
    }
  }) -> profk

ldply(list(R0=profR0,k=profk),.id='profile') -> profIF

ldply(list(det=profTM,stoch=profIF),.id='model') %>%
  saveRDS(file='profiles.rds')

closeCluster(cl)
mpi.quit()
```

Executing this code will result in the creation of several files, of which `profiles.rds` is the most important, containing as it does the results of all the parameter estimation.

Diagnostics

The codes in file `diagnostics.R` compute the diagnostics displayed in the paper. These computations are not very heavy, but can be accelerated using `doMC` if run on a multi-core workstation. In a directory containing `profiles.rds` (see above) and `ebola.R`, execute these codes with a command like

```
Rscript --vanilla diagnostics.R
```

Contents of file `diagnostics.R`:

```
library(pomp)
library(plyr)
library(reshape2)
library(magrittr)
options(stringsAsFactors=FALSE)

require(foreach)
require(doMC)
require(iterators)

source("ebola.R")

readRDS("profiles.rds") %>%
  ddply(~country+type+model,subset,loglik==max(loglik)) %>%
  subset(type=="raw"&model=="stoch") -> mles

time1 <- c(Guinea="2014-01-05",Liberia="2014-06-01",
```

```
          SierraLeone="2014-06-08",WestAfrica="2014-01-05")

registerDoMC(4)

foreach (mle=iter(mles,by='row'),.combine=rbind) %dopar%
  {
    country=as.character(mle$country)
    type=as.character(mle$type)
    M <- ebolaModel(country=country,type=type,
                    na.rm=TRUE,nstage=3,timestep=0.01)
    p <- unlist(subset(mle,select=-c(country,type,model,profile,
                                     loglik,loglik.se,
                                     nfail.min,nfail.max,conv,etime)))
    coef(M,names(p)) <- unname(unlist(p))

    t0 <- as.Date(time1[country])

    simulate(M,nsim=10,as.data.frame=TRUE,obs=TRUE,include.data=TRUE,seed=2186L) %>%
      mutate(date=t0+time*7,country=country,type=type)
    } %>% saveRDS(file="diagnostics-sim.rds")

foreach (mle=iter(mles,by='row'),.combine=rbind) %dopar%
  {
    country=as.character(mle$country)
    type=as.character(mle$type)
    M <- ebolaModel(country=country,type=type,
                    na.rm=TRUE,nstage=3,timestep=0.01)
    p <- unlist(subset(mle,select=-c(country,type,model,profile,
                                     loglik,loglik.se,
                                     nfail.min,nfail.max,conv,etime)))
    coef(M,names(p)) <- unname(unlist(p))

    probe(M,probes=list(probe.acf(var="cases",lags=1,type="correlation")),
          nsim=500,seed=1878812716L) %>%
      as.data.frame() -> pb
    pb %>% mutate(sim=rownames(pb),
                  data=ifelse(sim=="data","data","simulation"),
                  type=type,
                  country=country)
    } %>% saveRDS(file="diagnostics-probes.rds")

## Additional diagnostics

## Run probes for each country
## Custom probe: exponential growth rate
probe.trend <- function (y) {
  cases <- y["cases",]
  df <- data.frame(week=seq_along(cases),cases=cases)
  fit <- lm(log1p(cases)~week,data=df)
  unname(coef(fit)[2])
  }

foreach (mle=iter(mles,by='row'),.combine=rbind) %dopar%
  {
    country=as.character(mle$country)
    type=as.character(mle$type)
    M <- ebolaModel(country=country,type=type,
                    na.rm=TRUE,nstage=3,timestep=0.01)
    p <- unlist(subset(mle,select=-c(country,type,model,profile,
                                     loglik,loglik.se,
                                     nfail.min,nfail.max,conv,etime)))
    coef(M,names(p)) <- unname(unlist(p))

    ## remove an exponential trend, give residuals on the log scale
    dm <- model.matrix(lm(log1p(cases)~time,data=as.data.frame(M)))
    rm <- diag(nrow(dm))-dm%*%solve(crossprod(dm))%*%t(dm)
    detrend <- function (x) log1p(x)%*%rm

    probe(M,probes=list(
      probe.acf(var="cases",lags=1,type="correlation"),
      sd=probe.sd(var="cases",transform=log1p),
      probe.quantile(var="cases",prob=c(0.9)),
      d=probe.acf(var="cases",lags=c(1,2,3),type="correlation",
```

```
                 transform=detrend),
      trend=probe.trend),
      nsim=2000,seed=2186L
      ) %>% as.data.frame() -> pb
    pb %>% mutate(sim=rownames(pb),
                 kind=ifelse(sim=="data","data","simulation"),
                 type=type,
                 country=country)
    } %>%
  melt(id=c("country","type","kind","sim"),variable.name="probe") %>%
  arrange(country,type,probe,kind,sim) %>%
  saveRDS(file="diagnostics-addl-probes.rds")
```

Forecasting

The codes in file `forecasts.R` perform all the forecasting computations. In a directory containing `ebola.R`, `profiles.rds`, and `hosts`, a command like

```
mpirun -hostfile hosts -np 101 Rscript --vanilla forecasts.R
```

will result in the execution of these computations.

Contents of the file `forecasts.R`:

```
require(pomp)
require(plyr)
require(reshape2)
require(magrittr)
options(stringsAsFactors=FALSE)

set.seed(988077383L)

require(foreach)
require(doMPI)
require(iterators)

source("ebola.R")

horizon <- 13

foreach (country=c("SierraLeone"),.inorder=TRUE,.combine=c) %:%
  foreach (type=c("raw","cum"),.inorder=TRUE,.combine=c) %do%
  {
    M1 <- ebolaModel(country=country,type=type,
                     timestep=0.01,nstageE=3,na.rm=TRUE)
    M2 <- ebolaModel(country=country,type="raw",
                     timestep=0.01,nstageE=3,na.rm=TRUE)
    time(M2) <- seq(from=1,to=max(time(M1))+horizon,by=1)
    M3 <- ebolaModel(country=country,type="raw",
                     timestep=0.01,nstageE=3,na.rm=TRUE)
    time(M3) <- seq(from=max(time(M1))+1,to=max(time(M1))+horizon,by=1)
    timezero(M3) <- max(time(M1))
    list(M1,M2,M3)
    } -> models
dim(models) <- c(3,2,1)
dimnames(models) <- list(c("fit","det.forecast","stoch.forecast"),
                         c("raw","cum"),c("SierraLeone"))

noexport <- c("models")

## Weighted quantile function
wquant <- function (x, weights, probs = c(0.025,0.5,0.975)) {
  idx <- order(x)
```

```r
  x <- x[idx]
  weights <- weights[idx]
  w <- cumsum(weights)/sum(weights)
  rval <- approx(w,x,probs,rule=1)
  rval$y
  }

starts <- c(Guinea="2014-01-05",Liberia="2014-06-01",SierraLeone="2014-06-08")

cl <- startMPIcluster()
registerDoMPI(cl)

bake <- function (file, expr) {
  if (file.exists(file)) {
    readRDS(file)
    } else {
      val <- eval(expr)
      saveRDS(val,file=file)
      val
      }
  }

readRDS("profiles.rds") %>%
  ddply(~country+type+model,subset,loglik>max(loglik)-6,
        select=-c(conv,etime,loglik.se,nfail.min,nfail.max,profile)) -> mles

mles %>% melt(id=c("country","type","model"),variable.name='parameter') %>%
  ddply(~country+type+model+parameter,summarize,
        min=min(value),max=max(value)) %>%
  subset(parameter!="loglik") %>%
  melt(measure=c("min","max")) %>%
  acast(country~type~model~parameter~variable) -> ranges

mles %>% ddply(~country+type+model,subset,loglik==max(loglik),select=-loglik) %>%
  mutate(k=round(k,4),rho=round(rho,4),R0=round(R0,4),E_0=3*round(E_0/3)) %>%
  unique() %>%
  arrange(country,type,model) -> mles

### DETERMINISTIC MODELS

bake(file="forecasts_det.rds",{
  foreach (country=c("SierraLeone"),
           .inorder=TRUE,.combine=rbind) %:%
    foreach (type=c("raw","cum"),nsamp=c(1000,3000),
             .inorder=TRUE,.combine=rbind) %do%
    {

      params <- sobolDesign(lower=ranges[country,type,'det',,'min'],
                            upper=ranges[country,type,'det',,'max'],
                            nseq=nsamp)

      foreach(p=iter(params,by='row'),
              .inorder=FALSE,
              .combine=rbind,
              .noexport=noexport,
              .options.multicore=list(set.seed=TRUE),
              .options.mpi=list(chunkSize=10,seed=1568335316L,info=TRUE)
              ) %dopar%
        {
          M1 <- models["fit",type,country][[1]]
          M2 <- models["det.forecast",type,country][[1]]
          ll <- logLik(traj.match(M1,start=unlist(p)))
          x <- trajectory(M2,params=unlist(p))
          p <- parmat(unlist(p),20)
          rmeasure(M2,x=x,times=time(M2),params=p) %>%
            melt() %>%
            mutate(time=time(M2)[time],
                   period=ifelse(time<=max(time(M1)),"calibration","projection"),
                   loglik=ll)
        } %>%
        subset(variable=="cases",select=-variable) %>%
        mutate(weight=exp(loglik-mean(loglik))) %>%
        arrange(time,rep) -> sims
```

```
      ess <- with(subset(sims,time==max(time)),weight/sum(weight))
      ess <- 1/sum(ess^2)
      cat("ESS det",country,type,"=",ess,"\n")

      sims %>%
        ddply(~time+period,summarize,prob=c(0.025,0.5,0.975),
              quantile=wquant(value,weights=weight,probs=prob)) %>%
        mutate(prob=mapvalues(prob,from=c(0.025,0.5,0.975),
                              to=c("lower","median","upper"))) %>%
        dcast(period+time~prob,value.var='quantile') %>%
        mutate(country=country,type=type)
      }
  }) -> fc_tm

### STOCHASTIC MODEL

bake(file="forecasts_stoch.rds",{
  foreach (country=c("SierraLeone"),
           .inorder=TRUE,.combine=rbind) %:%
    foreach (type=c("raw","cum"),nsamp=c(200,200),
             .inorder=TRUE,.combine=rbind) %do%
    {

      params <- sobolDesign(lower=ranges[country,type,'stoch',,'min'],
                            upper=ranges[country,type,'stoch',,'max'],
                            nseq=nsamp)

      foreach(p=iter(params,by='row'),
              .inorder=FALSE,
              .combine=rbind,
              .noexport=noexport,
              .options.multicore=list(set.seed=TRUE),
              .options.mpi=list(chunkSize=1,seed=1568335316L,info=TRUE)
              ) %dopar%
        {
          M1 <- models["fit",type,country][[1]]
          M2 <- models["stoch.forecast",type,country][[1]]
          pf <- pfilter(M1,params=unlist(p),Np=2000,save.states=TRUE)
          pf$saved.states %>% tail(1) %>% melt() %>%
            acast(variable~rep,value.var='value') %>%
            apply(2,function (x) {
              setNames(c(x["S"],sum(x[c("E1","E2","E3")]),x["I"],x["R"]),
                       c("S_0","E_0","I_0","R_0"))}) -> x
          pp <- parmat(unlist(p),ncol(x))
          pp[rownames(x),] <- x
          simulate(M2,params=pp,obs=TRUE) %>%
            melt() %>%
            mutate(time=time(M2)[time],
                   period=ifelse(time<=max(time(M1)),"calibration","projection"),
                   loglik=logLik(pf))
        } %>% subset(variable=="cases",select=-variable) %>%
        mutate(weight=exp(loglik-mean(loglik))) %>%
        arrange(time,rep) -> sims

      ess <- with(subset(sims,time==max(time)),weight/sum(weight))
      ess <- 1/sum(ess^2)
      cat("ESS stoch",country,type,"=",ess,"\n")

      sims %>% ddply(~time+period,summarize,prob=c(0.025,0.5,0.975),
                     quantile=wquant(value,weights=weight,probs=prob)) %>%
        mutate(prob=mapvalues(prob,from=c(0.025,0.5,0.975),
                              to=c("lower","median","upper"))) %>%
        dcast(period+time~prob,value.var='quantile') %>%
        mutate(country=country,type=type)
      }
  }) -> fc_if

ldply(list(stoch=fc_if,det=fc_tm),.id='model') %>%
  ddply(~country,mutate,
        model=factor(model,levels=c("stoch","det")),
        date=as.Date(starts[unique(as.character(country))])+7*(time-1)) %>%
  saveRDS(file='forecasts.rds')
```

```r
closeCluster(cl)
mpi.quit()
```

References

Ionides, E. L., Nguyen, D., Atchadé, Y., Stoev, S. & King, A. A., 2015 Inference for dynamic and latent variable models via iterated, perturbed Bayes maps, *Proceedings of the National Academy of Sciences of the U.S.A.* **112**, 719–724.

King, A. A., Ionides, E. L., Bretó, C. M., Ellner, S., Kendall, B., Wearing, H., Ferrari, M. J., Lavine, M. & Reuman, D. C., 2010 *pomp: Statistical inference for partially observed Markov processes (R package)*.

Raue, A., Kreutz, C., Maiwald, T., Bachmann, J., Schilling, M., Klingmüller, U. & Timmer, J., 2009 Structural and practical identifiability analysis of partially observed dynamical models by exploiting the profile likelihood, *Bioinformatics* **25**, 1923–9.

WHO Ebola Response Team, 2014 Ebola virus disease in West Africa—the first 9 months of the epidemic and forward projections, *New England Journal of Medicine* **371**, 1481–1495, PMID: 25244186.
